# Effects of Yoga on Psychological Health, Quality of Life, and Physical Health of Patients with Cancer: A Meta-Analysis

**DOI:** 10.1155/2011/659876

**Published:** 2011-03-09

**Authors:** Kuan-Yin Lin, Yu-Ting Hu, King-Jen Chang, Heui-Fen Lin, Jau-Yih Tsauo

**Affiliations:** ^1^School and Graduate Institute of Physical Therapy, College of Medicine, National Taiwan University, 3F, No. 17 Xuzhou Road, Taipei 100, Taiwan; ^2^Department of Surgery, Cheng Ching General Hospital, 118 Sec. 3 Chung Kang Road, Taichung 407, Taiwan; ^3^Division of Physical Therapy, Department of Physical Medicine and Rehabilitation, National Taiwan University Hospital, No. 7 Chung-Shan S. Road, Taipei 100, Taiwan

## Abstract

Yoga is one of the most widely used complementary and alternative medicine therapies to manage illness. This meta-analysis aimed to determine the effects of yoga on psychological health, quality of life, and physical health of patients with cancer. Studies were identified through a systematic search of seven electronic databases and were selected if they used a randomized controlled trial design to examine the effects of yoga in patients with cancer. The quality of each article was rated by two of the authors using the PEDro Scale. Ten articles were selected; their PEDro scores ranged from 4 to 7. The yoga groups compared to waitlist control groups or supportive therapy groups showed significantly greater improvements in psychological health: anxiety (*P* = .009), depression (*P* = .002), distress (*P* = .003), and stress (*P* = .006). However, due to the mixed and low to fair quality and small number of studies conducted, the findings are preliminary and limited and should be confirmed through higher-quality, randomized controlled trials.

## 1. Introduction

Cancer is a leading cause of death worldwide. The disease accounted for 7.4 million deaths (or around 13% of all deaths worldwide) in 2004 [1]. Patients with cancer often have to deal with severe side effects and psychological distress during cancer treatment, which have a substantial impact on their quality of life (QOL) [2]. Among the most common symptoms of cancer and the results of treatments for cancer are pain [3], depression [4], and fatigue [5]. These symptoms may appear or persist, even after treatment ends.

In addition to physical symptoms, people with cancer nearly always experience considerable levels of psychological distress. Psychological health in cancer patients is defined by the presence or absence of distress as well as the presence or absence of positive wellbeing and psychological growth. It is determined by the balance between two classes of factors: the stress and burden posed by the cancer experience and the resources available for coping with this stress and burden [6].

Many patients with cancer use forms of complementary and alternative medicine (CAM) to help manage the effects of their illness [7]. CAM encompasses a broad array of heterogeneous treatments, ranging from herbal medicine to yoga [8]. According to a previous survey, approximately 21% of cancer survivors in the United States engaged in CAM practices, and the third most common CAM practice was yoga [9]. As an adjunct to conventional cancer therapies, the complementary therapies in question are used to improve quality of life through decreasing the adverse effects of anticancer treatments or through alleviating the symptoms of cancer [10]. 

Yoga, as a main component of the Mindfulness-Based Stress Reduction (MBSR) program [11], is a combination of breathing techniques, physical postures, and meditation that have been practiced as various styles of hatha yoga for over 5,000 years [12]. It has been used to lower blood pressure, reduce stress, and improve coordination, flexibility, concentration, sleep, and digestion [13]. It has also been used as a supplementary therapy for such diverse conditions as cancer [8], diabetes [14], asthma [15], and AIDS [16]. 

Some studies have specifically demonstrated potential psychological benefits of yoga in various clinical populations, including patients with depression [27–29], stress [30], and anxiety [31, 32]. However, the results of these studies need to be interpreted carefully since many of the published studies regarding yoga are small and lack meticulous design. It may also be difficult to compare studies that evaluate different patient populations. Only a few systematic and comprehensive reviews of scientific research on yoga for patients with cancer have been published [8]. Also, none of the previously published reviews addresses the quantitative magnitude of the identified effects.

The aim of this meta-analysis was to determine the effects of yoga on psychological health (i.e., anxiety, depression, distress, and stress), quality of life, and physical health of people with cancer. In contrast to previous reviews [8, 33, 34], this review used a meta-analytical approach to provide an effect size (standardized mean differences (SMDs)) for yoga on cancer-related symptoms.

## 2. Methods

### 2.1. Literature Search

We searched Medline, PubMed, PEDro, EMBASE, the Cochrane Library, PsycINFO (formerly PsychLit), and CEPS (a Chinese database) from January 1970 to July 2010 using the keywords cancer, oncology, yoga, mindfulness, stress reduction, psychological health, physical health, quality of life, and randomized controlled trials (RCT) for relevant studies in English and Chinese. The reference sections of relevant articles were also reviewed by the authors.

### 2.2. Study Selection and Characteristics

Two reviewers (Lin and Hu) independently evaluated the abstracts identified by our search. To be included in the final analysis, studies had to use a randomized control trial design to examine the effects of yoga or MBSR on psychological health, quality of life, and physical health of cancer patients. Studies were excluded if they did not provide pre and poststudy data that were needed to calculate an effect size (standardized mean differences). If there were multiple assessment time points, the time point of postintervention was chosen. If data of change scores were not reported, attempts were made to obtain data from the study authors by e-mail. In cases in which change scores and standard deviations (SDs) were not obtainable, the study was excluded.

### 2.3. Data Abstraction and Validity Assessment

Two authors (Lin and Hu) independently assessed the methodological quality of the studies. PEDro Scale [35] was applied to rate the quality of each article. Studies with PEDro scores scoring below 4 were considered to be of “poor” methodological quality, studies scoring between 4 and 5 were considered to be of “fair” quality, studies ranging from 6 to 8 were considered to be of “good” quality, and studies scoring 9-10 were considered to be of “excellent” quality [36]. PEDro has been shown to have acceptable validity and reliability in systematic reviews of randomized controlled trials [37, 38] and has been used in other studies [39–42]. When there were different scores between the two reviewers for any article, a consensus score was assigned after a comprehensive discussion.

### 2.4. Statistics

To evaluate the agreement of using the PEDro Scale, kappa statistic was calculated for measuring agreement between two authors. Values of kappa between 0.40 and 0.59 have been considered to reflect fair agreement, between 0.60 and 0.74 to reflect “good” agreement and 0.75 or more to reflect “excellent” agreement [43]. The inter-rater reliability of the total PEDro score was evaluated using type 2, 1 intraclass correlation coefficients (ICCs) in SPSS 13.0 [38]. Meta-analysis was conducted for comparisons between yoga or MBSR and control groups across studies and was analyzed using Review Manager 5 (RevMan5) software. 

Changes from preintervention assessment to postintervention assessment were obtained directly from the study results or calculated by determining the difference between the reported mean before and after the intervention. Continuous outcomes were analyzed using weighted mean differences when all studies measured outcomes on the same scale. Standardized mean differences were used when all scales were assumed to measure the same underlying symptom or condition but some studies measured outcomes on different scales [44]. Ninety-five percent confidence intervals were computed for all outcomes. 

Heterogeneity was explored by Cochrane's *Q* test and *I* ^2^. *I* ^2^ can be interpreted as the proportion of total variation observed between the studies attributable to differences between studies rather than sampling error (chance).   *I* ^2^ > 75% is considered to be a heterogeneous meta-analysis, and a random-effects model was used; otherwise, a fixed-effects model was used for homogenous meta-analysis. Statistical significance was set as *P* < .05, indicating that the effects differed significantly between the intervention and control groups.

Sensitivity analysis was conducted to investigate potential sources of heterogeneity and to determine how sensitive the final conclusions of the study are to the particular method or study design feature that was used [44]. If the effect and confidence intervals in the sensitivity analysis lead to the same conclusion as the primary meta-analysis value, the results are deemed robust. Sensitivity analyses were performed in this study by the delivery mode of intervention and the types of participants.

## 3. Results

### 3.1. Description of Studies

**Figure 1:**
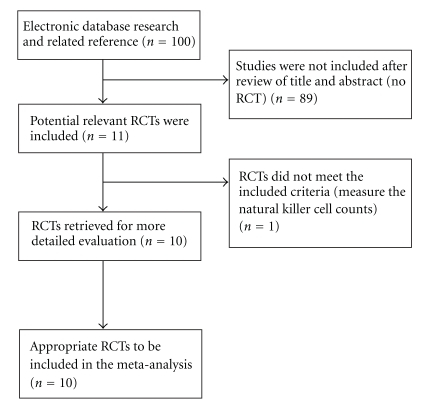
Flowchart detailing study selection.

A total of 100 articles were identified after searching by keywords. Following the exclusion process, a total of 11 randomized controlled trials met the inclusion criteria. From these 11 abstracts, 10 were identified as appropriate for further examination and the full articles were collected. The excluded study evaluated natural killer cell counts, rather than physical health, psychological health, and quality of life as its outcome measure [45]. Thus, 10 studies were finally included for analysis.  Figure 1 displays the flow diagram of study selection. Of the 10 studies, 7 assessed the effects of yoga for patients with breast cancer [17–23], 1 for patients with lymphoma [25], and 2 for mixed cancer population [24, 26].

The quality of the studies was assessed using the PEDro rating scale. Kappa statistics for agreement between reviewers on methodological quality was 0.80. The reviewers agreed on 90 of the 100 items (10 items for 10 studies) of the PEDro scale (90%). The intraclass correlation coefficients for interrater reliability of the total PEDro scores for individual raters were 0.94 (95% confidence interval (CI) 0.77~0.99). The median score for methodological quality of all included studies was 5 (PEDro scores ranged from 4 to 7). Of the 10 studies, 1 had a rating of 7 on a scale of 0–10 [20], 3 had a rating of 6 on a scale of 0–10 [18, 19, 21], 3 had a rating of 5 [17, 22, 26], and 3 had a rating of 4 [23–25] (Table 1). The characteristics of included studies are presented in Table 2.

The mean age of the participants across all the studies ranged from 43 to 58 years; 96% of participants were female and 4% male. The mean time since diagnosis ranged from 12 to 56 months. Studies included patients diagnosed with a variety of cancers, with 80% of participants having a breast cancer diagnosis and 63% of participants in early stage (Stages 0–II). 

For seven studies [17, 20, 22–26], participants in the control group were offered the opportunity to take part in the yoga or MBSR program after the study ended, and for the other three studies, the control intervention consisted of supportive counseling [18, 19, 21].

The style of yoga used and the duration and frequency of the yoga sessions varied among all studies. Integrated yoga consisted of a set of asanas (postures done with awareness), breathing techniques, including pranayama (voluntarily regulated nostril breathing), and meditation and yogic relaxation techniques with imagery were used by 3 of the studies [18, 19, 21]. The other 7 studies used restorative yoga [17], hatha yoga [22], Tibetan style yoga [25], and unspecified types of yoga [20, 23, 24, 26], respectively. Intervention duration ranged from 6 to 24 weeks, with 3 of the 10 studies [18, 20, 21] using yoga programs with 6-week duration, 3 studies [23, 25, 26] using 7-week programs, 1 study [24] using 8-week programs, 1 study [17] using 10-week programs, 1 study [22] using 12-week programs, and 1 study [19] using 24-week programs. Eight of the 10 studies [17, 20–26] used a group format for the intervention, and in most cases, home practice and homework were given to the participants, but only one of the studies [22] reported data on the adherence to home practice.

### 3.2. Outcome Analysis

#### 3.2.1. Psychological Health (Figure 2)

Eight of the 10 studies used anxiety as an outcome measure, but there was little consistency among studies with respect to which test was used to evaluate anxiety [18–21, 23–26]. Two studies used Hospital Anxiety and Depression Scale (HADS) [18, 21] as the primary measure of anxiety. Two studies used state trait anxiety inventory (STAI) [19, 20] anxiety state subscale; one study with lymphoma populations used Speilberger State Anxiety Inventory (STATE) (same as STAI) [25]. The other two studies used Profile of Mood States (POMS) [23, 26]. One study used Symptoms Checklist Revised (SCL-90-R) [24] to measure anxiety. Substantial heterogeneity was present in the comparison between yoga and control groups (*P* < .001, *I* ^2^ = 91%). The analyzed results revealed significantly greater improvement in yoga groups (*P* = .009). The pooled standardized mean difference was −0.76 (−1.34 to −0.19). 

Eight studies had at least one outcome measure for depression [17, 18, 20, 21, 23–26]. The outcome measures used to assess depression in the cancer populations were Hospital Anxiety and Depression Scale (HADS) [18, 21], Center for Epidemiologic Studies-Depression Scale (CES- D) [17, 20, 25], Profile of Mood States (POMS) [23, 26], and Symptoms Checklist Revised (SCL-90-R) [24]. The results showed significant improvements in the yoga groups (*P* = .002), and the standardized mean difference was −0.95 (−1.55 to −0.36).

Two of the 10 studies included distress as an outcome measure [22, 24]. Monti et al. used the Global Severity Index (GSI) of the SCL-90-R [24]. Another study used Distressed Mood Index to measure three domains of mood: anxious/sad, irritable, and confused [22]. The pooled standardized mean difference was −0.4 (−0.67 to −0.14) (*P* = .003). 

Although Cohen et al. also included distress as one of their outcome measures, they used the Impact of Events Scale (IES) [25], which is a different construct from the GSI and Distress Mood Index. IES is a self-report scale that measures the frequency of intrusive thoughts and avoidance in relation to patients' cancer. The results of this study showed no significant differences between the Tibetan Yoga group and wait-list control group in terms of the distress.

Several studies had outcome measures for the symptoms of stress [23, 26] or level of stress [18, 20, 21]. The Perceived Stress Scale was used to measure levels of stress. The Symptoms of Stress Inventory was used to measure physical, psychological, and behavioral responses to stressful situations. The overall effect of the yoga groups showed significantly greater improvement in stress level (*P* < .006), and the standardized mean difference was −0.95 (−1.63 to −0.27).

#### 3.2.2. Overall Quality of Life (Figure 3)

Three of the 10 studies measured QOL [17, 22, 23]. The 3 studies with outcome measurement data for quality of life outcomes used different measures, including the Functional Assessment of Cancer Therapy-Breast (FACT-B) [17], European Organization for Research and Treatment of Cancer Quality of Life Questionnaire Version 3.0 (EORTC QLQ-C30) [23], and Functional Assessment of Cancer Therapy: General (FACT-G) [22]. The quality of life of the yoga groups showed a trend toward more improvement (*P* = .06) than the control groups, and the standardized mean difference was −0.29 (−0.58 to 0.01).

#### 3.2.3. Physical Health (Figure 4)

The outcome measures used to assess physical health in the cancer populations were SF-12 health survey physical component summary (PCS) [17], physical composite score of the Medical Outcomes Study Short-Form Health Survey [20, 24] and The Functional Assessment of Cancer Therapy physical well-being subscale [22]. These measures are scored so that a high score indicates better physical functioning. Hence, the scores were multiplied by negative one so that the score direction would be the same as that of other variables. The improvement of physical health in the yoga groups were not significantly greater than that of the controls (*P* = .15), and the standardized mean difference was −0.16 (−0.37 to 0.06).

Four of the 10 studies included fatigue as an outcome measure [17, 22, 25, 26]. Two of 4 studies used the FACT-Fatigue [17, 22], one study used the Brief Fatigue Inventory (BFI) [25] to measure fatigue severity, and the other one used Profile of Mood States (POMS) [26]. The overall improvement of the yoga groups was not significant (*P* = .24), and the standardized mean difference was −0.15 (−0.39 to 0.09).

Only one study included fitness testing as one of its outcome measures; thus, this outcome was not included in the meta-analysis [23]. This was a pilot study to examine the physical and psychological benefits afforded by a 7-week yoga program for breast cancer survivors. In regard to the results of physical fitness, participants in both yoga and control groups showed some improvements over time.

### 3.3. Sensitivity Analysis

When we restricted the analysis to 8 studies [17, 20–26] that used a group format for intervention, no different findings were found among all the outcomes. Removal of any study with individual intervention did not significantly alter the heterogeneity or *P* value. Moreover, a sensitivity analysis identified no significant differences between studies that recruited women with breast cancer [17–22] and those that recruited participants deviated from this pattern [23–26].

## 4. Discussion

Previous reviews have reported that yoga is beneficial for people with cancer in managing symptoms such as fatigue, insomnia, mood disturbances and stress, and improving quality of life [33]. However, to our knowledge, until now the size of the effect has not been quantified. We conducted the first meta-analysis of studies investigating yoga interventions for patients with cancer. Data were extracted from 10 RCTs with a total of 762 participants with cancer. The results of our meta-analysis suggest that yoga may have positive effects on psychological health of cancer patients.

Many cancer patients experience cancer-related psychological symptoms, including mood disturbances, stress, and distress [33]. The results of our study revealed the efficacy of yoga on psychological health for cancer patients and are consistent with the result of a meta-analysis conducted by Ledesma and Kumano. [46], which indicated that mindfulness-based stress reduction programs may indeed be helpful for the mental health of cancer patients. Also, Carlson et al. showed that MBSR significantly improved the overall symptoms of stress in cancer patients and these improvements were maintained over a year of followup [47]. Thus, yoga may have long-term psychological effects for patients with cancer. 

However, the present findings do not address whether the psychological health benefits were attributable directly to yoga as a whole or the specific components of yoga, such as meditation and attention, in patients with cancer. Given that several yoga programs included meditation and relaxation with imagery, the positive results on psychological health might be obtained from these. Nevertheless, because of the nature of yoga interventions, it is impossible to control for placebo effects in investigations.

Although most RCTs reported anxiety, depression, and stress as outcome measures, the assessment tools used to measure their outcomes were inconsistent, which limits the generalization of the pooled results. Future research should focus on higher-quality trials with larger sample size in order to provide more precise estimates of the effects of yoga as a treatment.

Three of 10 RCTs reported quality of life outcomes, and the results showed a borderline difference between two groups. In contrast to the results of previous studies [48, 49], our results only showed a trend toward a small positive effect of yoga on quality of life. This may be due to the small sample size and the limited number of studies available for analysis. Moreover, different QOL measures were used across the studies, future study should develop and use standardized measures agreed by the research community.

Our results showed that the overall effects for physical health outcomes were statistically nonsignificant. According to the previous review [34], no significant differences were observed on the measure of physical health. Because of the limited number of studies and different measurement tools, the effects of yoga on physical health in people with cancer remain unclear. Furthermore, the studies included in the analysis used only the subscale of subjective questionnaires to report on physical health, meaning that our conclusions should be taken cautiously. Only one study [23] examined the effects of yoga on physical fitness; therefore, future study could include outcome measures that not only include subjective feelings in questionnaires but also include physical performance tests such as the 6-minute walk test, physical strength, endurance, and flexibility.

Moreover, our results did not show positive effects of yoga on fatigue. According to Sood's review [50], insufficient data exist at present to recommend any specific complementary and alternative medicine modality for cancer-related fatigue. Future studies with more participants and with a randomized clinical trial design should be conducted to investigate the effects of CAM interventions, including yoga, on cancer-related fatigue of cancer patients.

All studies included in the meta-analysis investigated participants with a diagnosis of cancer; however, the types of cancer varied among studies. Of the 10 included studies, 7 investigated breast cancer, 2 recruited mixed cancer populations, and 1 included patients with lymphoma. The result of Cohen's study on lymphoma [25] showed no significant differences between groups in terms of anxiety, depression, distress, or fatigue; thus, it has little influence on our result. Therefore, since the majority of studies focused on breast cancer, future research needs to examine the use of yoga among male cancer patients and female nonbreast cancer patients.

In addition, various factors are associated with the execution of the intervention such as yoga styles and treatment doses that may influence effect size. Four different styles of yoga were used among the included studies: restorative, integrated, hatha, and Tibetan. Treatment dose, including duration and frequency, and the adherence to yoga intervention and home practice may also affect treatment outcome. According to Carson's study on yoga for women with metastatic breast cancer [51], patients who practiced yoga longer on a given day were much more likely to experience less pain and fatigue and greater invigoration, acceptance, and relaxation on the next day. Future study needs to report adherence with the intervention protocol and the home practice to scrutinize the “dose-response” relationship between frequency and duration of yoga programs and changes on health outcomes.

All studies had inevitable limitations such as that it was not possible to blind subjects or therapists from group allocations in this type of empirical study. Therefore, the highest possible score that each study could get would be 8 out of 10 when using a PEDro scale or other types of quality criteria.

Considerable heterogeneity existed (*I* ^2^ > 70%) when the effects of yoga on anxiety, depression, and stress were compared with control groups. Several potential sources of clinical heterogeneity are to be considered when interpreting the results. These include population sample studied, treatment maneuver, and study design and methods [44]. Therefore, the random effect model was used as it addresses the variability that exists among studies.

The sensitivity analysis suggested that the effect of yoga was consistent across the intervention format and the types of cancer patients. As the literature search in this study was restricted to articles published in Chinese and English, this may introduce publication and language bias. Furthermore, this meta-analysis is limited due to the possibility of missing eligible unpublished or non-English studies and the fairly homogeneous studies included in the analysis. However, given the small number of studies included, the assessment of the heterogeneity or publication bias was difficult in this exposure.

## 5. Conclusion

In summary, our findings show potential benefits of yoga for people with cancer in improvements of psychological health. Because of the small number of studies having been conducted and the methodological limitations, the results should be regarded as preliminary and treated with caution. 

Our preliminary findings also provide practitioners with important information that yoga may be a possible adjunctive therapy for cancer patients to help manage psychological distress and to improve quality of life. Nevertheless, more attention must be paid to the physical effects of yoga and the methodological quality of future research, as well as to improve these areas in the future.

## Figures and Tables

**Figure 2:**
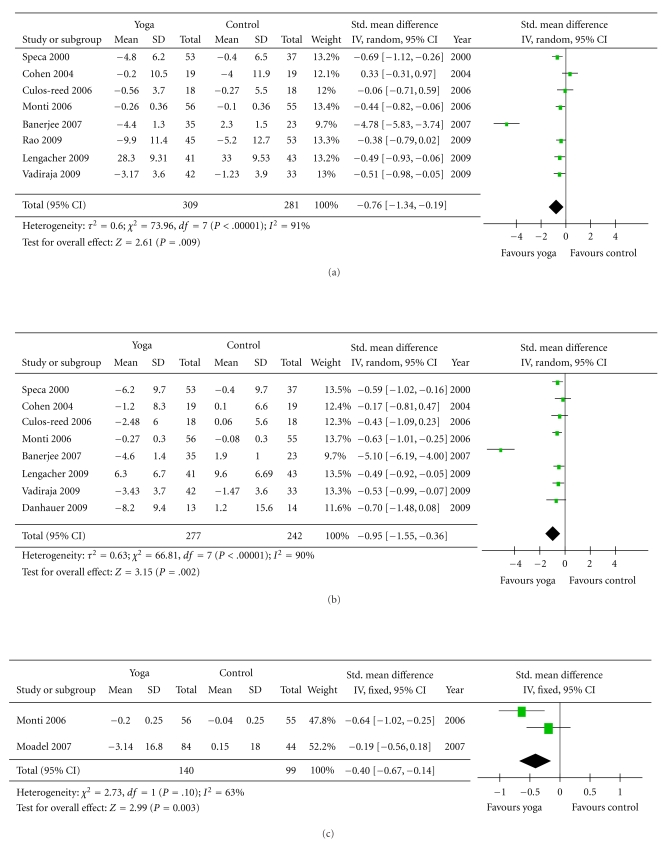

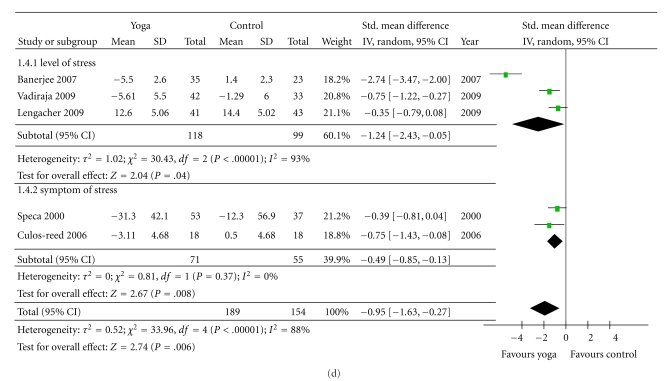
Comparison 1: yoga and control, psychological health, outcome: (a) Anxiety. (b) Depression. (c) Distress. (d) Stress.

**Figure 3:**
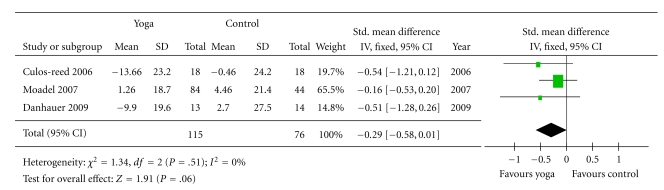
Comparison 2: yoga and control, outcome: Quality of life.

**Figure 4:**
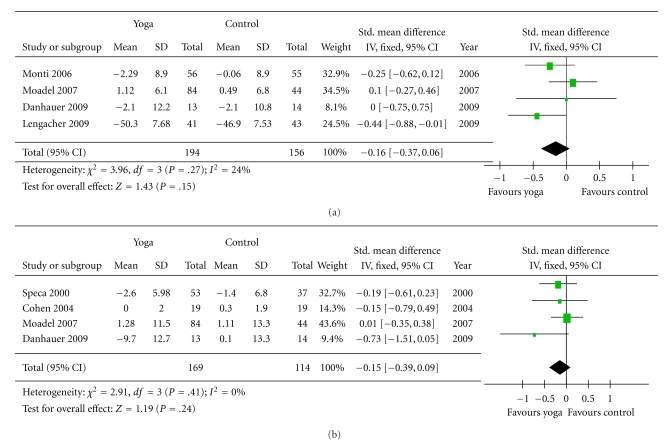
Comparison 3: yoga and control, outcome, physical health, outcome: (a) Physical health. (b) Fatigue.

**Table 1:** Methodological quality of analyzed studies.

PEDro criteria	Danhauer et al., 2009 [17]	Raghavendra et al., 2009 [18]	Rao et al., 2009 [19]	Lengacher et al., 2009 [20]	Banerjee et al., 2007 [21]	Moadel et al., 2007 [22]	Culos-reed et al., 2006 [23]	Monti et al., 2006 [24]	Cohen et al., 2004 [25]	Speca et al., 2000 [26]
(1) Eligibility criteria (not included in score)	1	1	1	1	1	1	0	1	1	1
(2) Random allocation	1	1	1	1	1	1	1	1	0	1
(3) Concealed allocation	0	1	1	0	1	0	0	0	0	0
(4) Baseline comparability	1	1	1	1	0	1	0	0	1	1
(5) Blind subjects	0	0	0	0	0	0	0	0	0	0
(6) Blind therapists	0	0	0	0	0	0	0	0	0	0
(7) Blind assessors	0	0	0	1	1	0	0	0	0	0
(8) Adequate followup	0	1	0	1	1	0	1	0	1	0
(9) Intention-to-treat analysis	1	0	1	1	0	1	0	1	0	1
(10) Between-group comparisons	1	1	1	1	1	1	1	1	1	1
(11) Point estimates and variability	1	1	1	1	1	1	1	1	1	1

Total	5/10	6/10	6/10	7/10	6/10	5/10	4/10	4/10	4/10	5/10

**Table 2:** Characteristic of included studies.

Author, year	No. of participants	Age, mean ± SD	Yoga type	Frequency	Duration, week	Outcome measures	Results
Danhauer et al., 2009 [17]	44	55.8 ± 9.9	Restorative yoga: asanas (postures done with awareness), pranayama (voluntarily regulated nostril breathing), and savasana (deep relaxation)	75 min weekly	10	QOL (SF-12 and FACT_B), fatigue (FACT-Fatigue), depression (CES-D), positive and negative affect (PANAS), and spirituality (FACIT-Sp), sleep quality (PSQI)	Group differences favoring yoga group in mental health, depression, positive affect, and spirituality. Significant baseline*group interactions in negative affect and emotional well-being.
Raghavendra et al., 2009 [18]	88	46.0 ± 9.1	Integrated yoga: asanas, pranayama, meditation, and yogic relaxation	1 hr, 3 sessions weekly	6	Anxiety and depression (HADS), level of stress (PSS)	Significant between group differences on anxiety (*P* < .001), depression (*P* = .002), perceived stress (*P* < .001), salivary cortisol (*P* = .009), and pooled mean cortisol (*P* = .03). significant positive correlation between morning salivary cortisol level and anxiety and depression
Rao et al., 2009 [19]	98	NA	Integrated yoga: asanas, pranayama, meditation, and yogic relaxation	60 min daily	24	Anxiety (STAI)	Overall decrease in both self-reported state anxiety (*P* < .001) and trait anxiety (*P* = .005)
Lengacher et al., 2009 [20]	84	57.5 ± 9.4	MBSR: gentle yoga	2 hr weekly	6	Fear of recurrence (Concerns about Recurrence Scale, anxiety (STAI), depressive symptoms (CES-D), optimism (Life Orientation Test), perceived stress (PSS), QOL (SF), social support (MOSS), spirituality	Significant between group differences on levels of depression, anxiety, fear of recurrence, energy, physical functioning, and physical role functioning (two-sided *P* < .05)
Banerjee et al., 2007 [21]	68	44.0 ± 1.4	Integrated yoga: asanas, pranayama, nidra (guided relaxation with imagery)	90 min weekly	6	Anxiety and depression (HADS), level of stress (PSS), DNA damage	Significant between group differences on HADS score, mean PSS, and DNA damage (*P* < .001)
Moadel et al., 2007 [22]	128	54.8 ± 9.9	Hatha yoga: physical stretches, breathing, and meditation	1.5 hr weekly	12	QOL (FACT-G), spiritual well-being (FACIT-Sp), fatigue (FACIT-Fatigue), distress (Distressed Mood Index)	Significant between group differences on social well-being (*P* < .0001). For patients not receiving CT during intervention, significant between group differences on overall QOL, emotional well-being, social well-being, spiritual well-being, and distress mood
Culos-reed et al., 2006 [23]	38	51.1 ± 10.3	Yoga: asanas, shevasana (relaxation)	75 min	7	Anxiety and depression (POMS), QOL (EORTC QLQ-C30), symptom of stress (SOSI), physical activity (LSI), fitness (grip strength, flexibility, and Rockport Walking Test)	Significant differences between groups at post-intervention in global QOL, emotional function, diarrhea, and tension (*P* < .05).
Monti et al., 2006 [24]	111	53.6 ± 11.5	MBAT: gentle yoga	2.5 hr weekly	8	Psychological distress and stress-related somatic complaints (SCL-90-R), Health-related QOL (SF-36)	Significant between group differences on symptoms of distress (*P* < .001), mental composite of SF-36 (*P* = .025), general health (*P* = .008), mental health (*P* < .001), social functioning (*P* = .048), and vitality (*P* = .01)
Cohen et al., 2004 [25]	39	51	Tibetan yoga: controlled breathing, mindfulness, postures from Tsa lung (channels and vital breath), Trul khor (magical wheel)	weekly	7	Distress (IES), anxiety (STATE), depression (CES-D), fatigue (BFI), sleep disturbances (PSQI)	Significant between group differences on sleep disturbance scores (*P* < .004), sleep quality (*P* < .02), sleep latency (*P* < .01), sleep duration (*P* < .03), use of sleep medications (*P* < .02). no significant differences between groups in terms of intrusion, state anxiety, depression, or fatigue
Speca et al., 2000 [26]	90	51	MBSR: gentle yoga	90 min weekly	7	Anxiety and depression (POMS), stress-related symptoms (SOSI)	Significant between group differences on total mood disturbance, subscales of depression, anxiety, anger, confusion, vigor, and symptoms of stress.

Abbreviations: QOL: quality of life; HADS: Hospital Anxiety and Depression Scale; PSS: Perceived Stress Scale; STAI: state trait anxiety inventory; SF-12: The 12-Item Short Form Health Survey; FACT-B: Functional Assessment of Cancer Therapy-Breast; FACT-Fatigue: Functional Assessment of Cancer Therapy-Fatigue; FACIT-Sp: Functional Assessment of Chronic Illness Therapy-Spirituality; CES-D: Center for Epidemiologic Studies Depression Scale; PSQI: Pittsburgh sleep quality inventory; PANSA: positive and negative affect schedule; FACT-G: The Functional Assessment of Cancer Therapy-General; FACIT-Fatigue: Functional Assessment of Chronic Illness Therapy-Fatigue; POMS: profile of mood states; SOSI: symptoms of stress inventory; EORTC QLQ-C30: European Organization for Research and Treatment of Cancer Quality of Life Questionnaire Version 3.0; LSI: The Leisure Score Index; IES: Impact of Events Scale; STATE: Speilberger State Anxiety Inventory; BFI: Brief Fatigue Inventory; NA: not available; MBSR: mindfulness-based stress reduction; SF: Medical Outcomes Studies Short-Form General Health Survey; MOSS: Medical Outcomes Social Support Survey; MBAT: mindfulness-based art therapy; SCL-90-R: Symptoms Checklist Revised; SF-36: Medical Outcomes Study Short-Form Health Survey; CT: chemotherapy; min: minute; hr: hour.

## References

[1] Cancer. http://www.who.int/mediacentre/factsheets/fs297/en/index.html.

[2] Luebbert K, Dahme B, Hasenbring M (2001). The effectiveness of relaxation training in reducing treatment-related symptoms and improving emotional adjustment in acute non-surgical cancer treatment: a meta-analytical review. *Psycho-Oncology*.

[3] Christo PJ, Mazloomdoost D (2008). Cancer pain and analgesia. *Annals of the New York Academy of Sciences*.

[4] Walker J, Sharpe M (2009). Depression care for people with cancer: a collaborative care intervention. *General Hospital Psychiatry*.

[5] Barsevick AM, Newhall T, Brown S (2008). Management of cancer-related fatigue. *Clinical Journal of Oncology Nursing*.

[6] Andrykowski MA, Lykins E, Floyd A (2008). Psychological health in cancer survivors. *Seminars in Oncology Nursing*.

[7] Duncan MD, Leis A, Taylor-Brown JW (2008). Impact and outcomes of an iyengar yoga program in a cancer centre. *Current Oncology*.

[8] Smith KB, Pukall CF (2009). An evidence-based review of yoga as a complementary intervention for patients with cancer. *Psycho-Oncology*.

[9] Fouladbakhsh JM, Stommel M (2010). Gender, symptom experience, and use of complementary and alternative medicine practices among cancer survivors in the U.S. cancer population. *Oncology Nursing Forum*.

[10] Ernst E (2009). Complementary and alternative medicine (CAM) and cancer: the kind face of complementary medicine. *International Journal of Surgery*.

[11] Smith JE, Richardson J, Hoffman C, Pilkington K (2005). Mindfulness-based stress reduction as supportive therapy in cancer care: systematic review. *Journal of Advanced Nursing*.

[12] Hadi N (2007). Effects of hatha yoga on well-being in healthy adults in Shiraz, Islamic Republic of Iran. *Eastern Mediterranean Health Journal*.

[13] Barnes PM, Powell-Griner E, McFann K, Nahin RL (2004). Complementary and alternative medicine use among adults: United States, 2002. *Advance Data*.

[14] Aljasir B, Bryson M, Al-Shehri B (2009). Yoga practice for the management of type II diabetes mellitus in adults: a systematic review. *Evidence-Based Complementary and Alternative Medicine*.

[15] Manocha R, Marks GB, Kenchington P, Peters D, Salome CM (2002). Sahaja yoga in the management of moderate to severe asthma: a randomised controlled trial. *Thorax*.

[16] Fritts M, Crawford CC, Quibell D (2008). Traditional Indian medicine and homeopathy for HIV/AIDS: a review of the literature. *AIDS Research and Therapy*.

[17] Danhauer SC, Mihalko SL, Russell GB (2009). Restorative yoga for women with breast cancer: finding from a randomized pilot study. *Psycho-Oncology*.

[18] Raghavendra RM, Vadiraja HS, Nagarathna R (2009). Effects of a Yoga program on cortisol rhythm and mood states in early breast cancer patients undergoing adjuvant radiotherapy: a randomized controlled trial. *Integrative Cancer Therapies*.

[19] Rao MR, Raghuram N, Nagendra HR (2009). Anxiolytic effects of a yoga program in early breast cancer patients undergoing conventional treatment: a randomized controlled trial. *Complementary Therapies in Medicine*.

[20] Lengacher CA, Johnson-Mallard V, Post-White J (2009). Randomized controlled trial of mindfulness-based stress reduction (MBSR) for survivors of breast cancer. *Psycho-Oncology*.

[21] Banerjee B, Vadiraj HS, Ram A (2007). Effects of an integrated yoga program in modulating psychological stress and radiation-induced genotoxic stress in breast cancer patients undergoing radiotherapy. *Integrative Cancer Therapies*.

[22] Moadel AB, Shah C, Wylie-Rosett J (2007). Randomized controlled trial of yoga among a multiethnic sample of breast cancer patients: effects on quality of life. *Journal of Clinical Oncology*.

[23] Culos-Reed SN, Carlson LE, Daroux LM, Hately-Aldous S (2006). A pilot study of yoga for breast cancer survivors: physical and psychological benefits. *Psycho-Oncology*.

[24] Monti DA, Peterson C, Shakin Kunkel EJ (2006). A randomized, controlled trial of mindfulness-based art therapy (MBAT) for women with cancer. *Psycho-Oncology*.

[25] Cohen L, Warneke C, Fouladi RT, Rodriguez MA, Chaoul-Reich A (2004). Psychological adjustment and sleep quality in a randomized trial of the effects of a Tibetan yoga intervention in patients with lymphoma. *Cancer*.

[26] Speca M, Carlson LE, Goodey E, Angen M (2000). A randomized, wait-list controlled clinical trial: the effect of a mindfulness meditation-based stress reduction program on mood and symptoms of stress in cancer outpatients. *Psychosomatic Medicine*.

[27] Uebelacker LA, Epstein-Lubow G, Gaudiano BA, Tremont G, Battle CL, Miller IW (2010). Hatha yoga for depression: critical review of the evidence for efficacy, plausible mechanisms of action, and directions for future research. *Journal of Psychiatric Practice*.

[28] Pilkington K, Kirkwood G, Rampes H, Richardson J (2005). Yoga for depression: the research evidence. *Journal of Affective Disorders*.

[29] Shapiro D, Cook IA, Davydov DM, Ottaviani C, Leuchter AF, Abrams M (2007). Yoga as a complementary treatment of depression: effects of traits and moods on treatment outcome. *Evidence-Based Complementary and Alternative Medicine*.

[30] Mohan J (1996). Stress management and yoga. *International Journal of Psychology*.

[31] Kirkwood G, Rampes H, Tuffrey V, Richardson J, Pilkington K (2005). Yoga for anxiety: a systematic review of the research evidence. *British Journal of Sports Medicine*.

[32] Gupta N, Khera S, Vempati RP, Sharma R, Bijlani RL (2006). Effect of yoga based lifestyle intervention on state and trait anxiety. *Indian Journal of Physiology and Pharmacology*.

[33] DiStasio SA (2008). Integrating yoga into cancer care. *Clinical Journal of Oncology Nursing*.

[34] Bower JE, Woolery A, Sternlieb B, Garet D (2005). Yoga for cancer patients and survivors. *Cancer Control*.

[35] Chiang YC, Yeh CH, Wang KWK, Yang CP (2009). The experience of cancer-related fatigue in Taiwanese children. *European Journal of Cancer Care*.

[36] Foley NC, Teasell RW, Bhogal SK, Speechley MR (2003). Stroke rehabilitation evidence-based review: methodology. *Topics in Stroke Rehabilitation*.

[37] Macedo LG, Elkins MR, Maher CG, Moseley AM, Herbert RD, Sherrington C (2010). There was evidence of convergent and construct validity of Physiotherapy Evidence Database quality scale for physiotherapy trials. *Journal of Clinical Epidemiology*.

[38] Maher CG, Sherrington C, Herbert RD, Moseley AM, Elkins M (2003). Reliability of the PEDro scale for rating quality of randomized controlled trials. *Physical Therapy*.

[39] Thomas MJ, Simpson J, Riley R, Grant E (2010). The impact of home-based physiotherapy interventions on breathlessness during activities of daily living in severe COPD: a systematic review. *Physiotherapy*.

[40] Waller B, Lambeck J, Daly D (2009). Therapeutic aquatic exercise in the treatment of low back pain: a systematic review. *Clinical Rehabilitation*.

[41] May S, Johnson R (2008). Stabilisation exercises for low back pain: a systematic review. *Physiotherapy*.

[42] Li WC, Chen YC, Yang RS, Tsauo JY (2009). Effects of exercise programmes on quality of life in osteoporotic and osteopenic postmenopausal women: a systematic review and meta-analysis. *Clinical Rehabilitation*.

[43] Higgins JP, Green S Cochrane handbook for systematic reviews of interventions, version 5.0.0. http://www.cochrane-handbook.org/.

[44] Helewa A, Walker J (2000). *Critical Evaluation of Research in Physical Rehabilitation*.

[45] Rao RM, Telles S, Nagendra HR (2008). Effects of yoga on natural killer cell counts in early breast cancer patients undergoing conventional treatment. *Medical Science Monitor*.

[46] Ledesma D, Kumano H (2009). Mindfulness-based stress reduction and cancer: a meta-analysis. *Psycho-Oncology*.

[47] Carlson LE, Speca M, Faris P, Patel KD (2007). One year pre-post intervention follow-up of psychological, immune, endocrine and blood pressure outcomes of mindfulness-based stress reduction (MBSR) in breast and prostate cancer outpatients. *Brain, Behavior, and Immunity*.

[48] Ulger O, Yagli NV (2010). Effects of yoga on the quality of life in cancer patients. *Complementary Therapies in Clinical Practice*.

[49] Speed-Andrews AE, Stevinson C, Belanger LJ, Mirus JJ, Courneya KS (2010). Pilot evaluation of an iyengar yoga program for breast cancer survivors. *Cancer Nursing*.

[50] Sood A, Barton DL, Bauer BA, Loprinzi CL (2007). A critical review of complementary therapies for cancer-related fatigue. *Integrative Cancer Therapies*.

[51] Carson JW, Carson KM, Porter LS, Keefe FJ, Shaw H, Miller JM (2007). Yoga for women with metastatic breast cancer: results from a pilot study. *Journal of Pain and Symptom Management*.

